# Antioxidant potential of microencapsulated bioactive extracts from durian fruit’s pulp, seed, and peel as food additives

**DOI:** 10.3389/fnut.2026.1822584

**Published:** 2026-05-29

**Authors:** Victor Velazquez-Martinez, Patricia Cabrales-Arellano, Efren Delgado, Paweł Paśko, Govinda Sapkota, Julian Quintero-Quiroz, Juan Rodrigo Laguna-Camacho, Shela Gorinstein

**Affiliations:** 1 Department of Family and Consumer Sciences, Food Science and Technology, New Mexico State University, Las Cruces, NM, United States; 2 Facultad de Ingeniería Mecánica Eléctrica, Universidad Veracruzana, Poza Rica, Mexico; 3 Department of Biology, Eastern New Mexico University, Portales, NM, United States; 4 Department of Food Chemistry and Nutrition, Faculty of Pharmacy, Jagiellonian University Medical College, Kraków, Poland; 5 Facultad de Ciencias Alimentarias y Farmacéuticas, Universidad CES, Medellin, Colombia; 6 Institute for Drug Research, School of Pharmacy, Faculty of Medicine, The Hebrew University of Jerusalem, Jerusalem, Israel

**Keywords:** antioxidant, DSC, microcapsules, polyphenols, SEM, spray-drying, UHPLC

## Abstract

**Background:**

Durian pulp consumption is growing rapidly worldwide, but so is the waste it generates: seeds and peels. This study investigates the Total Phenolic Content (TPC) and antioxidant activity of extracts and microencapsulated extracts from the main components of the durian fruit: seed (Dse), pulp (Dpu), and peel (Dpe).

**Methods:**

The TPC was evaluated by the Folin-Ciocalteu method, and the antioxidant activity was measured using the DPPH, FRAP, and ABTS methods. The phenolic compounds profile was obtained by UPLC-MS/MS. Microencapsulation of durian extracts was achieved by spray drying using gum arabic and polydextrose as encapsulating agents. A Differential Scanning Calorimeter analyzed the thermostability of microcapsules, while the bioaccessibility of phenolic compounds was measured using an in vitro digestion test.

**Results:**

The seed extracts (18 mg GAE/g DW) showed higher TPC than pulp and peel extracts (1.7 and 6.5 mg GAE/ g DW, respectively). The seed extracts showed the highest antioxidant capacity across all assays (DPPH: 9.9, FRAP: 9.7, ABTS: 9.8 mg TE/g DW) and were strongly correlated with TPC (r = 0.73–0.91, *p* < 0.001). UPLC results showed that flavonoids (52%) are the predominant polyphenols, followed by phenolic acids (25%), lignans and coumarins (16%), and tannins (6%). Microencapsulation of durian extracts was achieved by spray drying under the optimal conditions of 53.1% gum arabic and 46.9% polydextrose, determined through an experimental design. The microcapsules of the Dse, Dpu, and Dpe extracts were thermostable, whereas the free extracts showed degradation over the temperature range from −4 to 200 °C. The *in vitro* digestion test showed 69.8–99.7% bioaccessibility of phenolic compounds across the oral, gastric, and intestinal phases, depending on the fruit component, where Dse microcapsules exhibited the highest bioaccessibility.

**Conclusion:**

The results of this study demonstrate the promising potential of valorizing bioactive compounds from durian fruit and its waste through extraction and microencapsulation for food and pharmaceutical applications.

## 1 Introduction

The durian (*Durio zibethinus L.*) fruit, also known as “The king of all fruits,” is characterized by its unique flavor and odor and is native to Southeast Asia. This fruit is mainly produced in Indonesia, Malaysia, the Philippines, and Thailand (1, 2). Thailand and Malaysia are the major exporters of durian fruit. While China accounts for the majority of global durian imports, the market for durian in the United States is growing rapidly. According to the Food and Agriculture Organization of the United Nations (FAO), global durian trade has increased significantly, rising over tenfold from 2003 to 2022 (3). Over 300 tons of durian were imported into the United States in the first quarter of 2024 (4).

Durian fruit, with the pulp as the commonly consumed part, is recognized for its health benefits, credited to its antioxidant properties (1, 5, 6). Major bioactive compounds reported in durian fruits are polyphenols, such as tannins, phenolic acids, flavonoids, and carotenoids. Carotenoids such as *α*-carotene, *β*-carotene, β-cryptoxanthin, lycopene, lutein, and zeaxanthin have been identified in the pulp. Durian fruit is also rich in vitamins, including B1, B2, A, C, and E, as well as minerals such as calcium, phosphorus, potassium, and iron (7).

Durian fruit extracts have been reported to exhibit excellent anti-proliferative and anticancer activities (7, 8). The significant antioxidant activity of durian seed, peel, and pulp extract is attributed to the different bioactive compounds present in them (8). The antidiabetic and anti-hypercholesterolemic properties of durian peel extract have also been reported (8).

The edible pulp (aril) accounts for 15–30% of the total fruit weight, while seeds and the peel account for 60–75% (9). The amount of seed and peel waste generated after retrieving the durian pulp is substantial, leaving ample opportunity to valorize it.

According to Putra et al., 866,000 tons of durian fruit waste are generated each year in Southeast Asia alone, and the figure is likely higher worldwide (10). Conventional disposal of durian fruit waste is not environmentally friendly and can cause landfill pollution, air pollution, soil degradation, and water pollution (11). Thus, innovative approaches to valorizing durian fruit waste will not only add to the economy but also reduce environmental hazards (11, 12). These wastes have been reported to be a good source of bioactive compounds (5).

Among the various ways to valorize durian fruit and its waste, the extraction of bioactive compounds and the production of microcapsules for use as food additives constitute a practical solution. Microencapsulated bioactive extracts have potential applications in functional foods. Salami, ice cream, and other baked goods can be fortified with microcapsules containing durian’s bioactive extracts, providing consumers with health benefits. Microencapsulation provides physical barriers that limit oxygen diffusion and light exposure to the core bioactive compounds, while controlled release can be achieved through matrix erosion, diffusion through the wall material, or triggered release by pH or enzymatic action during gastrointestinal digestion (13, 14).

Microencapsulation, a technique that coats ingredients such as antioxidant compounds with an outer core matrix of natural or synthetic biodegradable agents such as polymer or lipids, provides an opportunity to increase the shelf-life of antioxidant compounds that otherwise would degrade because of their sensitivity to factors such as oxidation, light, and temperature (13). Nevertheless, the properties of coating materials such as protective properties against external factors (light, temperature, moisture, and oxidation), compatibility with core material, controlled release, contribution to the stability and improved solubility of the core material, safety and biocompatibility are important in the production of microcapsules to be used as food additive and in pharmaceuticals (14, 15). In addition, the thermal stability of microcapsules and the bioaccessibility of bioactive compounds are also important for the successful utilization/incorporation in food applications.

This study aimed to analyze and compare the Total Phenolic Content (TPC) and antioxidant activities of extracts from durian seed, pulp, and peel to assess the bioaccessibility of microencapsulated bioactive compounds after *in vitro* digestion and to gain a deeper understanding of the valorization of durian fruit and its waste. Therefore, the microencapsulation of bioactive extracts from durian components using spray drying with gum arabic and polydextrose was investigated. Thermal stability and in vitro digestion of microcapsules produced with the optimal microencapsulating agent ratio were also studied.

## 2 Materials and methods

### 2.1 Sample preparation

Mature Monthong durian fruit from Thailand was purchased and separated into its three main components: seed (Dse), pulp (Dpu), and peel (Dpe). The Dpu was blended and placed in aluminum trays. The Dse and Dpe were cut into pieces. All samples were stored in the freezer at −18 °C for 36 h. Lyophilization was selected for Dpu to preserve its heat-sensitive bioactive compounds and volatile profile, whereas convection oven-drying at 50 °C was applied to Dse and Dpe due to their harder, fibrous matrices, which are more resistant to thermal degradation and benefit from extended low-temperature drying for efficient moisture removal. The Dpu sample was freeze-dried at −80 °C for 36 h (FreeZone Labconco, Kansas City, MO, USA), and the Dse and Dpe samples were oven-dried in a convection oven cyclone series (Bakers Pride, TN, US) at 50 °C for 48 h. After drying, Dse and Dpe samples were passed through a 1 mm sieve using a Wiley Mill 4 (Thomas Scientific, NJ, US), and Dpu samples were passed through a 1 mm sieve using a CT 293 Cyclotec mill (FOSS, Hillerød, DK). The Wiley Mill was used for the fibrous Dse and Dpe matrices, while the Cyclotec mill was employed for the lyophilized Dpu due to its brittle, porous texture. Both mills were operated with 1 mm sieves to ensure comparable final particle sizes across all samples. After reducing the particle size, samples were stored at −4 °C until further analysis.

### 2.2 Chemicals

2,4,6-tripyridyl-s-triazine (TPTZ), Potassium chloride ACS reagent 99% from Millipore Sigma (Burlington, MA, USA), 2,2′-Azino-bis(3-ethylbenzothiazoline-6-sulfonic acid) diammonium salt, Potassium persulfate, Calcium chloride dihydrate, 99 + %, ACS reagent, Ammonium carbonate, Potassium dihydrogen phosphate 98 + %, Pancreatin, porcine pancreas from Thermo Scientific Chemicals (Waltham, MA), Gallic acid, (±)-6-hydroxy-2,5,7,8-tetramethylchromane-2-carboxylic acid (Trolox) from ACROS Organics (Geel, Belgium); sodium carbonate anhydrous ACS reagent, Pepsin ≥ 250 units/mg, *α*-Amylase 11 units/mg from Sigma-Aldrich (St. Louis, MO, USA); Folin–Ciocalteu from MP Biomedicals (LLC, Irvine, CA, USA), Sodium chloride from Fisher Chemical (Pittsburgh, PA, USA), Bile salts from Spectrum Chemical (New Brunswick, NJ, USA).

### 2.3 Extraction of bioactive compounds from durian components

The bioactive compounds were extracted from durian Dpu, Dpe, and seed Dse by mixing 1 g of each sample with 10 mL of 60% ethanol, following previous studies on bioactive compound extraction (16–19) where the ethanol concentration was selected to provide further understanding. All mixtures were continuously stirred at 50 °C in an orbital shaker MaxQ HP Incubated Tabletop Orbital Shaker (ThermoFisher Scientific, Waltham, MA, USA) for 4 h at 120 rpm. The samples were then centrifuged for 10 min at 3,500 rpm at 22 °C (5810R; Eppendorf North America, Enfield, CT, USA). All supernatants were vacuum-filtered through grade 1 filter paper (Whatman plc, Maidstone, UK). The filtered samples (free bioactive extracts) were analyzed for TPC and antioxidant activity on the same day.

### 2.4 Total phenolic content (TPC)

The TPC was analyzed by the Folin–Ciocalteu method (17). Hundred microliter of each sample was diluted with 900 μL of deionized (DI) water and then mixed with 0.5 mL of 50% Folin–Ciocalteu reagent diluted with DI water. The mixture was incubated in the dark at room temperature for 5 min, then 1.5 mL of 20% sodium carbonate was added and vortexed. After adding 7 mL of DI water, the solutions were incubated at 75 °C for 10 min in a water bath (StableTemp, Cole-Parmer, Vernon Hills, IL). After incubation, absorbance was measured at 760 nm in a Genesys 10S UV–Vis spectrophotometer (Thermo Fisher Scientific) against a blank. TPC values were expressed as gallic acid equivalents (GAE) using the gallic acid calibration curve (*y* = 0.7098x + 0.04313, *R*^2^ = 0.99) with a concentration range of 0.0625 to 2 mg/mL.

### 2.5 Antioxidant activity

#### 2.5.1 Ferric reducing antioxidant power (FRAP)

The assay followed a method used in a previous study (20). Briefly, the FRAP reagent was freshly prepared by mixing 10 mM TPTZ dissolved in 40 mM HCl, 300 mM acetate buffer at pH 3.6, and 20 mM FeCl_3_·6H_2_O previously dissolved in DI water in a 1:10:1 volume/volume/volume (v/v/v) ratio. After, 100 μL of each sample extract was mixed with 900 μL of DI water and 2 mL of preheated FRAP reagent at 37 °C. Following incubation in the water bath (StableTemp, Cole-Parmer, Vernon Hills, IL, USA) at 37 °C for 30 min in the dark, the absorbance was read at 593 nm against a blank (1 mL DI water mixed with 2 mL FRAP reagent) using a spectrophotometer. A Trolox standard calibration curve was developed (*y* = 4.227x + 0.101, *R*^2^ = 0.99) with a concentration range of 0.008 to 1 mg/mL, and the FRAP values were expressed as milligram Trolox equivalent per gram of dry weight (mg TE/g DW).

#### 2.5.2 DPPH (2,2-diphenyl-1-picrylhydrazyl)

The scavenging activity was measured following the DPPH free radical method (21). The DPPH reagent powder was dissolved in ethanol to a final concentration of 0.1 mM. An aliquot of 100 μL of each sample extract was vigorously mixed with 3 mL of DPPH reagent and incubated at room temperature in the dark for 15 min. The absorbance was measured at 517 nm in a spectrophotometer against an ethanol blank. A Trolox standard calibration curve was developed (*y* = −0.510x + 0.544, R^2^ = 0.99) over a concentration range of 0.13 to 1.00 mg/mL to convert DPPH values to mg TE/g DW.

#### 2.5.3 ABTS [2,2′-azino-bis-(3-ethylbenzothiazoline-6-sulfonic acid)]

The ABTS radical-scavenging activity was evaluated using the methodology described by (22), with minor modifications. An ABTS stock solution (7 mM) was prepared and mixed with 2.45 mM potassium persulfate at a 1:0.5 ratio. The stock solution was incubated in the dark for 23 h prior to the sample analysis. An aliquot of the stock solution was diluted with ethanol until an absorbance of 1.00 ± 0.01 at 734 nm was achieved. Furthermore, 1 mL of the ABTS reagent solution was mixed with 10 μL of the sample/standard. A minute after incubation in the dark, absorbance was recorded at 734 nm against the blank (1 mL ABTS reagent with 10 μL ethanol). A Trolox standard calibration curve (*y* = 101.48x + 0.241, *R*^2^ = 0.99) with a concentration range from 0.008 to 1.00 mg/mL was used to express ABTS values as mg TE/g DW.

### 2.6 Phenolic compounds profile

#### 2.6.1 Sample preparation

Each sample of durian fruit (Dse, Dpu, and Dpe) extract was precisely weighed (~50 mg) into a 2.0 mL Eppendorf tube. Twelve microliter of methanol/water (7:3, v/v) solution was added per mg of sample. After vortexing for 15 min at room temperature, the samples were subsequently centrifuged for 3 min at 12,000 rpm at 4 °C. The clear supernatants were collected and filtered through a 0.22 μm membrane for the following assays.

#### 2.6.2 UPLC conditions

Aliquots of 2 μL from the filtered supernatants were injected for UPLC-MRM/MS analysis on an ExionLC AD UHPLC system coupled to a Sciex QTRAP mass spectrometer operated in the multiple reaction monitoring (MRM) mode. An Agilent SB-C18 UPLC column was used for the separation. A mobile phase composed of 0.1% formic acid in water and acetonitrile was used for binary solvent gradient elution (0–9 min, 5–95% B; 9–10 min, 95% B; 10–11.1 min, 95–5% B; 11.1–14 min, 5% B). The mobile-phase flow rate was 0.35 mL/min. The column temperature was maintained at 40 °C (Creative Proteomics, Shirley, NY, USA).

#### 2.6.3 ESI-Q TRAP-MS/MS

The ESI source operation parameters were as follows: source temperature 500 °C; ion spray voltage (IS) 5,500 V (positive ion mode)/−4,500 V (negative ion mode); ion source gas I (GSI), gas II (GSII), and curtain gas (CUR) were set at 50, 60, and 25 psi, respectively.

### 2.7 Microencapsulation of bioactive compounds

The Dse, Dpu, and Dpe extracts were reduced by rotoevaporation (water bath temperature = 60 °C, RPM = 80, and pressure ~200 mbar) to a final solid content of 5 °Brix. Gum arabic, used in a previous study (20), and polydextrose, a complex carbohydrate known for its probiotic effects and non-toxicity (23, 24), were chosen as microencapsulation agents. The core-to-coat ratio was 1:5 weight/weight (w/w). The mixture experimental design contained 8 treatment combinations of the encapsulating agents, with each agent contributing from 0 to 100%. Every treatment combination was mixed with 10 mL of Dse reduced extract, adjusted to 250 mL with DI water, and homogenized for 1 min at 7000 rpm (Polytron Ch-6010 PT 10–35 Kinematica, Bohemia, NY, USA). The dependent variable was the percentage of each encapsulating agent at a fixed inlet temperature of 125 °C, air supply pressure of 0.8 bar, outlet temperature ranged from 65 to 75 °C and the feed pressure (0.9–1.2 bar) was controlled to maintain a relative humidity in the chamber of less than 12% in an FT80 tall-form spray dryer (Armfield, Clarksburg, NJ, USA). The response variables were encapsulation efficiency (EE) (%), TPC (mg GAE eq/200 mg microcapsules), and ABTS (mg TEAC/200 mg microcapsules). The EE was calculated following the method described in our previous study (25). Briefly, to measure TPC, an amount of microcapsules (200 mg) was dissolved with a mixture (2 mL) of methanol, acetic acid, and DI water in a ratio of 50, 8, and 42%, respectively. To measure the surface phenolic content (SPC), 200 mg of microcapsules was mixed with 2 mL of methanol-ethanol (1:1). Both TPC and SPC mixtures were vortexed for 1 min and centrifuged at 3500 rpm for 5 min, and measured by the Folin Ciocalteu method. The EE was calculated using the equation:

$$\mathrm{EE}\;(\%)=\frac{\mathrm{TPC}-\mathrm{SPC}}{\mathrm{TPC}}x\;100$$

The optimal treatment combination was then used to microencapsulate the reduced extracts of Dpu and Dpe.

### 2.8 Evaluation of moisture content and water activity of microcapsules

The moisture content and water activity of the microcapsules were measured using a Sartorius MA160 moisture analyzer (Bohemia, NY, USA) and an Aqualab 4TE water activity meter (Pullman, WA, USA).

### 2.9 Scanning electron microscopy of microcapsules

A small amount of spray-dried microcapsules (7–8 mg) from each sample was transferred to the surface of aluminum stubs with a carbon tab. The images were retrieved using an SU-7000 Field Emission Scanning Electron Microscope (Hitachi High-Tech America Inc., Schaumburg, IL, USA). The voltage and magnification were 15 kV and 1,000x, respectively.

### 2.10 Thermal stability by differential scanning calorimeter (DSC)

The thermal stability analysis was conducted using Iridium as a standard on a DSC 3 + analyzer (Mettler Toledo LLC, Columbus, OH, USA). The lyophilized free extracts, encapsulating agents, encapsulated extracts, and empty capsules were weighed (7–8 mg) into 40 μL aluminum pans and sealed separately. Each pan was perforated and heated from −4 to 200 °C at 20 °C/min under Nitrogen (50 mL/min).

### 2.11 *In vitro* digestion of microcapsules

The bioaccessibility of bioactive compounds was measured using an *in vitro* digestion test described in a previous study, with minor modifications (26). The oral phase was conducted by mincing 1 g of microencapsulated either Dse, Dpu, or Dpe extracts with 1.4 mL of simulated salivary fluid. Afterward, 0.2 mL of alpha-amylase (1,500 U/mL), 10 μL of 0.3 M CaCl_2_, and 390 μL of Milli-Q water were added to the mixture, which was then incubated in a shaker incubator at 37 °C and 120 rpm for 2 min. For the gastric phase, 3 mL of simulated gastric fluid was added to the previous oral bolus, followed by 640 μL of pepsin (25,000 U/mL), 2 μL of CaCl_2_, 80 μL of 1 M HCl to reach a pH of 3.0, and 275 μL of Milli-Q water. The mixture was maintained in an orbital shaker at 37 °C and 120 rpm for 2 h. Afterward, 4.4 mL of simulated intestinal fluid was added to the gastric chyme along with 2 mL of pancreatin solution (800 u/mL), 1 mL of bile salts (160 mM), 16 μL of 1 M NaOH, and 524 μL of Milli-Q water. The mixture was incubated for 2 h at 37 °C in an orbital shaker at 120 rpm. After incubation, all tubes were centrifuged at 3000 g for 10 min, and TPC was measured in all supernatants for comparison with the TPC values before *in vitro* digestion.

### 2.12 Statistical analysis

A multivariate analysis was performed to compare TPC and antioxidants; the contrasts were performed using the Nonparametric Comparison of Multivariate Samples (npmv) RStudio package. A paired *t*-test was conducted to analyze differences in TPC before and after in vitro digestion. A mixture randomized experimental design with a quadratic model was used for microencapsulation. RStudio 2024.12.0 and DesignExpert V.13.05.0 were used for statistical analysis.

## 3 Results and discussion

### 3.1 Total phenolic content

The TPC varied across durian components, yielding a higher TPC (18 mg GAE/g DW) in Dse (*p* < 0.05), followed by Dpe (6.5 mg GAE/g DW) and Dpu (1.7 mg GAE/g DW), respectively (Table 1). Dse from our study exhibited 4.9 times higher TPC values (18 mg GAE/g DW) than the 3.67 mg GAE/g DW reported for seed extracts (27). Our results showed 1.7 to 2.1 times lower TPC in the pulp extracts than in the concentrations (3.83 and 3.09 mg GAE/g DW) reported in previous studies (28, 29). The TPC (6.5 mg GAE/g DW) in Dpe from our study was lower by 1.2 to 2.8 times than the TPC (8–18.5 mg GAE/g DW) reported by Sang-Ngam et al. (30).

**Table 1:** Total phenolic content and antioxidant activity of fresh extracts from durian “Monthong” components.

Method	Dse	Dpu	Dpe
TPC (mg GAE/g DW)	18.0 ± 0.75*^A^	1.7 ± 0.23^B^	6.5 ± 0.41^C^
DPPH (mg TE/g DW)	9.9 ± 0.02^A^	2.9 ± 0.23^B^	9.9 ± 0.05^C^
FRAP (mg TE/g DW)	9.7 ± 0.18^A^	2.8 ± 0.08^B^	7.4 ± 0.0.22^C^
ABTS (mg TE/g DW)	9.8 ± 0.15^A^	1.8 ± 0.23^B^	7.1 ± 0.0.56^C^

Dse = durian seed, Dpu = durian pulp, Dpe = durian peel. *Standard deviation. Contrasts: same letters for no differences in a row.

The differences in the values depend on the extraction method, extraction time, temperature, solvent type, and solvent concentration. For instance, methanol has been reported by some studies to extract better than ethanol (19, 31, 32), but it is also more toxic to human consumption. In our study, the extraction method was performed once, and TPC and antioxidant activity were measured in the resulting extract rather than concentrating and resuspending it. In addition, the TPC values from this study, where Dpu showed the lowest TPC and Dpe the highest, are consistent with the trend reported by Charoenphun’s study (5). The significant differences in TPC values between Charoenphun’s study and this study may be due to differences in fruit maturity. Charoenphun et al. used unripe fruit, whereas our study used mature fruit.

Moreover, the high generation of durian waste mentioned earlier (866,000 tons in Southeast Asia alone) provides an excellent source for bioactive extraction. For instance, according to our findings (18 and 6.5 mg GAE/g DW for Dse and Dpe, respectively), durian seeds and peel will yield over 4 tons of bioactive compounds each, making them readily available for industrial applications.

### 3.2 Antioxidant activity

The antioxidant capacities of Dse, Dpu, and Dpe, evaluated using DPPH, FRAP, and ABTS assays, are presented in Table 1. Among the extracts, Dse showed the greatest antioxidant activity across all assays, followed by Dpe and Dpu. Both Dse and Dpe exhibited identical DPPH values of 9.9 mg TE/g DW. A marked difference in antioxidant capacity was observed between the Dse and Dpu extracts. The FRAP value (9.7 mg TE/g DW) of Dse was ≈ 3.5 times that of the Dpu extract, while the ABTS value (9.0 mg TE/g DW) of Dse was ≈ 5.4 times that of the Dpu extract.

The results of this study on antioxidant activity follow the same trend as TPC, indicating a strong positive relationship between TPC and measured antioxidant activities, including DPPH (*r* = 0.73, *p* < 0.0001), FRAP (*r* = 0.91, *p* < 0.001), and ABTS (*r* = 0.90, *p* < 0.001). This finding is consistent with other studies reporting a strong positive correlation (*r* = 0.88–0.98) between total phenolic content and antioxidant activity across various fruits, including durian (18, 33, 34).

Although studies on durian fruit phenolic content and antioxidant activity in reference to individual components: seed, peel, and pulp in a single study are very scarce, different studies have evaluated the antioxidant activity of durian fruits using different methods and quantification standards. Mature durian fruit flesh has been reported to have DPPH, FRAP, and ABTS of 11.9 μmol Trolox/g DW, 6.79 μmol Trolox/g DW, and 21.0 μmol Trolox/g DW, respectively (29). Deng et al. (27) reported FRAP values of 7.85 μmol Fe(II)/g for durian seeds. DPPH and FRAP values in peel extracts from different durian varieties ranged from 21.80–38.88% scavenging at 250 μg/mL and 177.54–3.96 μmol Trolox Equivalent/g, respectively (30). According to the findings of Charoenphun et al., the antioxidant activity of durian fruit varies by variety and the antioxidant assay method employed (5). The study reported that the seed had the lowest ABTS and nitric oxide metal-ion chelating activity among the varieties “Monthong” and “Chanee.” However, the superoxide scavenging activity was highest in the seed extracts of both varieties. Nonetheless, the potential health benefits of phenolic extracts from each component of durian fruit can be further explored as individual phenolic compounds vary with the fruit component (Table 2), which could exhibit varying potential for scavenging different types of free radicals.

**Table 2:** Phenolic compounds identified by UPLC-MS/MS.

Phenolic compound	Dse	Dpu	Dpe	Class I
Catechin	593.7	85.9	1407.4	Flavonoids
Quercitrin	20.6	23.8	256.6	Flavonoids
Epicatechin	527.5	73.2	1350.3	Flavonoids
Cynaroside	20.2	13.2	18.6	Flavonoids
Trifolin	5.2	24.6	25.7	Flavonoids
Hesperetin	3.7	0.6	1.3	Flavonoids
Anacardic acid	112.7	1.0	32.1	Phenolic acids
p-Coumaric acid	106.6	125.7	172.9	Phenolic acids
Sinapic acid	15.1	61.0	118.6	Phenolic acids
Ferulic acid	154.2	720.8	86.6	Phenolic acids
Caffeic acid	94.2	158.9	100.2	Phenolic acids
Gallic acid	177.1	5.3	316.0	Phenolic acids
Decursinol	20.9	7.3	89.3	Lignans and Coumarins
Scopoletin	152.5	5.4	319.7	Lignans and Coumarins
Fraxidin	31.2	28.3	56.3	Lignans and Coumarins
Fraxetin	2176.0	1326.7	466.3	Lignans and Coumarins
Procyanidin C1	139.5	42.8	54.3	Tannins
Procyanidin C2	101.3	26.9	7.9	Tannins
Procyanidin B1	1516.8	143.5	534.8	Tannins
Cinnamtannin D1	13.8	18.9	12.2	Tannins

Values are expressed as mg/L. Dpe = durian peel, Dpu = durian pulp, Dse = durian seed. The table shows a portion of the identified phenolic compounds.

### 3.3 Phenolic compounds profile

**Figure 1:**
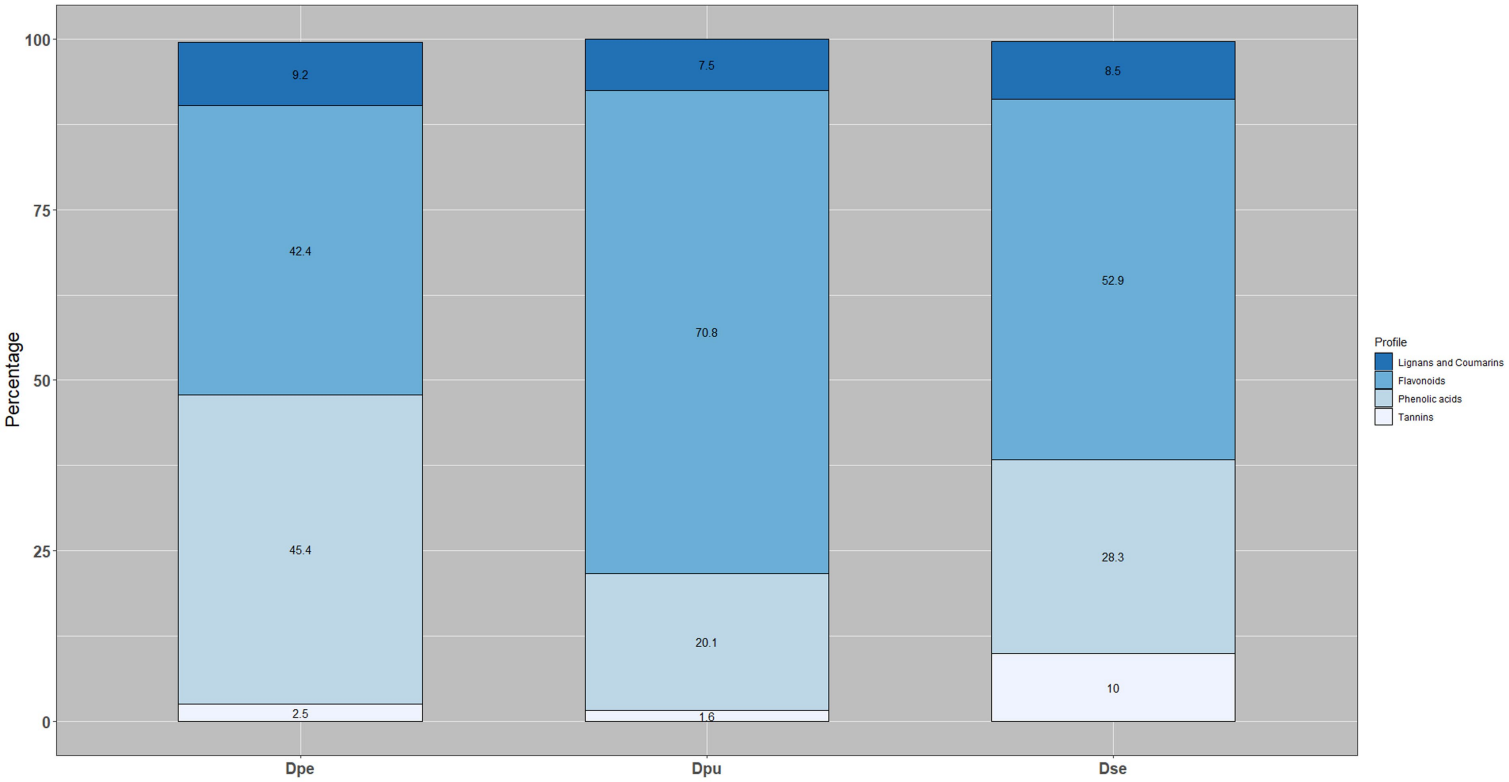
Phenolic compounds present in durian ethanolic extracts. The percentage contribution is from each of the three components analyzed in this study: seed, shell, and pulp. Data was retrieved from UPLC-MS/MS.

The profile obtained was intended to identify the majority of phenolic compounds in the samples rather than quantify selected or common ones, as limited information was available on durian waste at the time of this study. UPLC-MS/MS identified 876 compounds (Supplementary Table S1) in each durian component, of which 453 were flavonoids. Dpu exhibits the major contribution to its total phenolic compounds from flavonoids (70.8%), while Dse accounted for 53%, followed by Dpe with 42.6% (Figure 1). Phenolic acids provides 45.4% of the total compounds to Dpe, 28.3% to Dse, and 20.1% to Dpu. In addition, lignans and coumarins contribute 9.2, 8.5, and 7.5% to Dpe, Dse, and Dpu, respectively, while tannins and some unidentified phenolic compounds contribute less than 10% across durian components (Figure 1). Dse exhibited larger peak areas for several phenolic compounds (Supplementary Table S1) than Dpe and Dpu, which could explain the difference in antioxidant activity reported in this study, and is comparable to the findings in other fruit samples (27, 35). Moreover, the larger peak areas in Dse, followed by Dpe and Dpu, are expected, as plants concentrate polyphenols mainly in seeds and peel as defense mechanisms against pathogens and stress, or to inhibit germination (36).

Table 2 includes some of the phenolic compounds identified in durian components. Catechin, Quercitrin, and Sinapic acid identified in this study were also identified by He et al. using UHPLC-LTQ-OrbiTrap-MS/MS in Dpe (37). In other studies, Hesperetin, Gallic acid, Caffeic acid, and Ferulic acids were reported in Dpu (1, 38–40). Among the 453 flavonoids identified, Catechin and Epicatechin had higher peak areas in Dse extracts, followed by Dpe and Dpu. Catechin and Epicatechin have been reported to have positive effects on neurological diseases and obesity (41) and on carcinogenic cells and bacteria (42).

Table 2 lists some of the phenolic acids and tannins identified (222 and 40 compounds, respectively, in the Supplementary Table S1). Various compounds identified have been reported to have potential health benefits. For example, Anacardic acid has anticancer activity, and its antitumor effect was studied by encapsulating it in microcapsules made of sodium hyaluronate to enhance its antitumor activity (43). In addition, Anacardic acid was incorporated into a chitosan biofilm to create a food packaging film with antimicrobial activity to prevent browning in apple slices (44). Furthermore, Tannins were also used as antibrowning agents in asparagus and lettuce, inhibiting tyrosinase activity (45). Lastly, 144 different compounds belonging to the lignans class were identified in durian components. Lignans and coumarins exhibit anti-inflammatory effects by blocking the signaling proteins involved in the Janus Kinase, nuclear factor, and activator protein signaling pathways (46) or by modulating signaling pathways such as the COX-2 pathway (47). For example, Fraxetin, a coumarin identified in durian components in this study, exhibited an anti-growth effect on HaCat cells, which are related to skin cancer (48). In addition, Fraxetin acted as a coadjuvant to reduce the toxicity of doxorubicin, a cancer treatment medication (49).

### 3.4 Microencapsulation of bioactive compounds

The lyophilized powder from Dse was used to run the mixture experimental design for microencapsulation, as this sample exhibited higher TPC and antioxidant activity than Dpe and Dpu. Table 3 shows the quadratic equation coefficients for each response variable, obtained to fit the second-order model. The interaction effect (AB) of gum arabic and polydextrose was significant (*p* < 0.05), meaning that the interaction between polydextrose and gum arabic affected the EE. It was observed that the EE decreased as the percentage of gum arabic in the coating mixture decreased. The AB was also significant (*p* < 0.05) for TPC per 200 mg of microcapsules, following the same behavior as EE. For ABTS, neither factor A nor factor B nor the interaction AB was significant. The non-significant effect of the encapsulating agent ratio on ABTS values suggests that ABTS-reactive compounds in the durian seed extract may be less sensitive to the encapsulation matrix composition, possibly because the radical scavenging mechanisms measured by ABTS (primarily electron transfer) are less affected by the physical entrapment efficiency than those measured by TPC and EE, which depend more directly on the integrity of the wall material system. The desirability index was 0.9 for the optimal solution, 53.1% gum arabic, and 46.9% polydextrose. The results from the optimal treatment (EE = 93.3%, TPC/200 mg microcapsules = 13.6 TE, ABTS = 25.1 TE) fell within the 95% confidence interval, thereby validating the model.

**Table 3:** Coefficients of the effect of the mixture ratio between gum arabic and polydextrose on the encapsulation efficiency (EE), total phenolic content (TPC), and the antioxidant activity measured by the ABTS method.

Model term	EE %		TPC mg GAE/200 mg		ABTS mg TE/200 mg	
	Coefficients	*p*-value	Coefficients	*p*-value	Coefficients	*p*-value
A	98.84	0.0003*	12.67	0.0251*	24.33	0.1268
B	93.83	0.0002*	12.86	0.8068	28.90	0.2268
AB	10.66	0.002*	9.85	0.0094*	23.71	0.0868

The encapsulating agents were used to encapsulate the extracted bioactive compounds from durian seed by spray drying. A = gum arabic, B = polydextrose. *Significant at α = 0.05 level.

The significant interaction (AB) between gum Arabic and polydextrose on EE and TPC can be attributed to their complementary physicochemical properties. Gum Arabic, with its amphiphilic arabinogalactan protein fraction (~10%), provides effective emulsification and film-forming capacity at the droplet interface, while polydextrose contributes to matrix rigidity and low hygroscopicity. At the optimal ratio (53.1:46.9), the synergistic effect likely results from enhanced matrix density and reduced porosity, limiting phenolic compound migration to the capsule surface during spray drying.

### 3.5 Evaluation of moisture content and water activity of microcapsules

The average moisture content was 5.21–5.67%, and the water activity ranged from 0.19 to 0.29 across Dse, Dpu, and Dpe microcapsules, with no significant differences. Moisture content below 7% prevents water diffusion and oxygen access to the microcapsule core, while low water activity reduces microcapsule chemical and microbial degradation and increases shelf-life (50), thus ensuring the storage of spray-dried microcapsules. These values are also below the critical thresholds for Maillard reactions and lipid oxidation, supporting long-term stability of the encapsulated phenolic compounds.

### 3.6 Scanning electron microscopy of microcapsules

**Figure 2:**
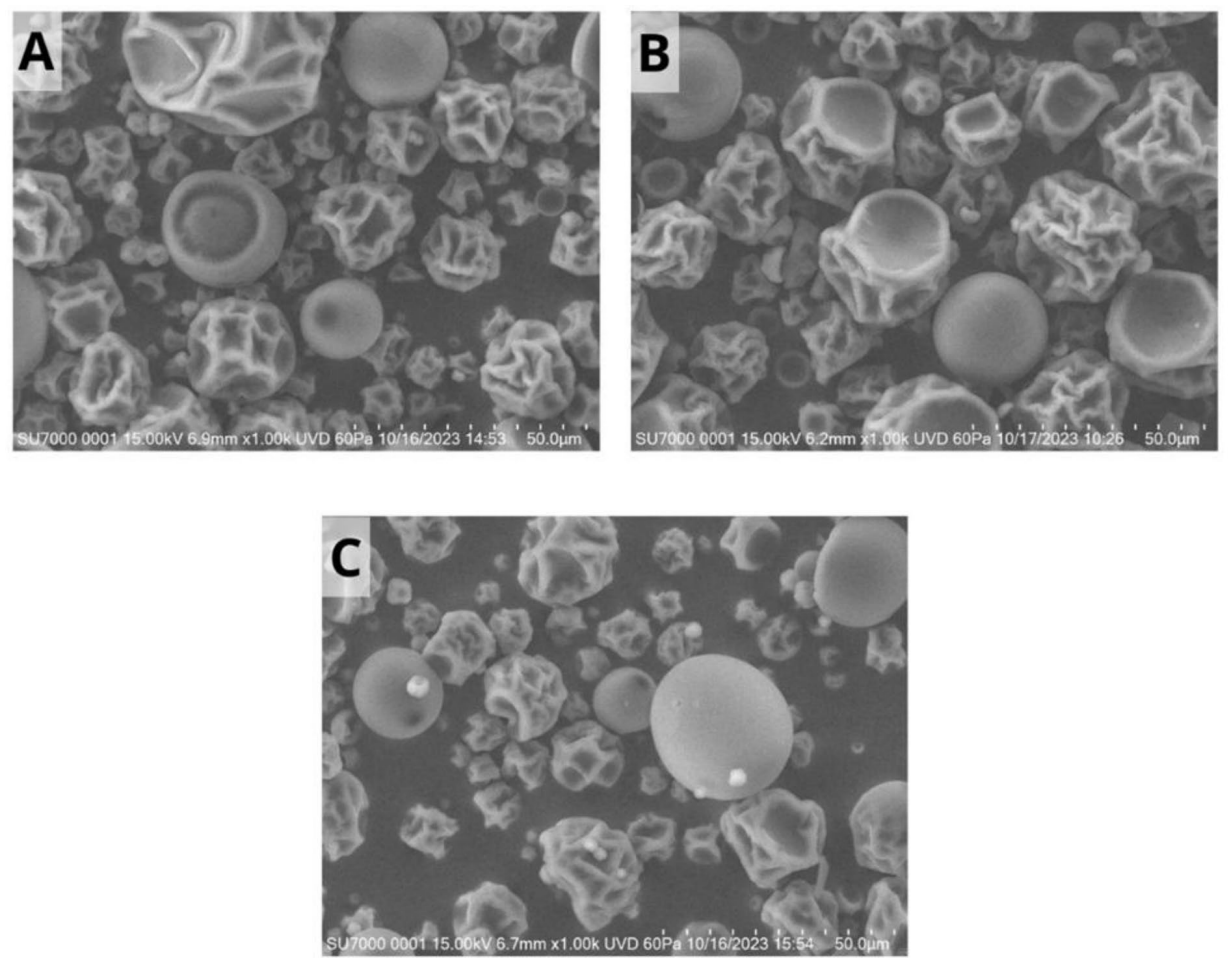
Morphology images from SEM of spray-dried microcapsules with gum arabic and polydextrose as wall materials and extracts from different components of durian as core material **(A–C)**. **(A)** = durian seed, **(B)** = durian pulp, **(C)** = durian peel.

The experimental design for microencapsulation was conducted using lyophilized powders from Dse extracts, selected for their higher TPC and antioxidant activity. The optimal treatment from the Dse experimental design was used to encapsulate the lyophilized extracts from Dse, Dpu, and Dpe. The ‘deflated’ appearance and surface indentations (Figure 2) are usually due to the spray dryer’s rapid drying and cooling. In addition, water vapor inside the capsules escapes during cooling, creating a wrinkled surface on the microcapsules (19, 50, 51). The indented microcapsules do not show cracks in any of the three images, indicating high EE and no coalescence, due to the film-forming capacity of the gum arabic-polydextrose mixture. A few large spherical capsules were found, and some showed ruptures, which could lead to oxidation of the core material, although this did not affect EE.

The microcapsules of all three durian components ranged in size from 2 to 45 μm. The size difference can be attributed to protein, which can form a layer on the capsules during drying, leading to interactions with the coating materials and contributing to particle size (51, 52). In this case, gum arabic, as a wall material, can serve as a protein source, as previous studies have reported that it contains approximately 10% arabinogalactan protein (53, 54). Although the particle size of the microcapsules varied, the findings on the size range of this study align with the food industry’s needs for powders (52), and the surface morphology observed in our samples would help to increase the solubility of microcapsules during rehydration (55).

### 3.7 Thermal stability

**Figure 3:**
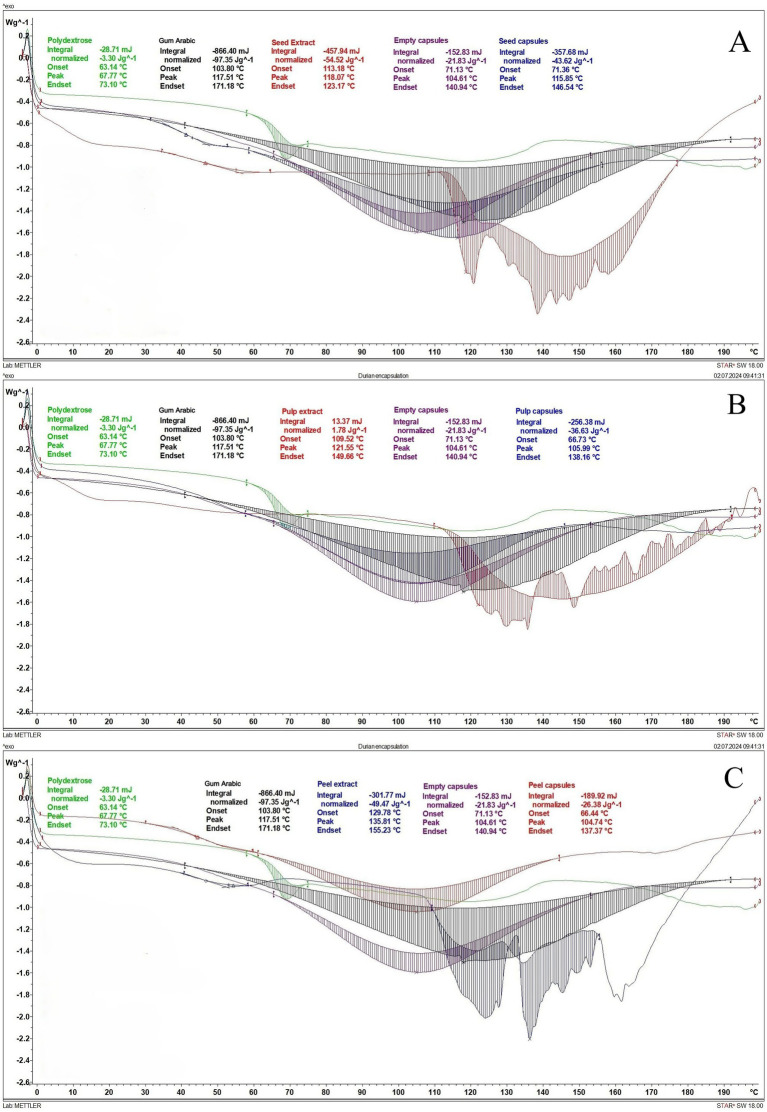
DSC curves of polydextrose, gum arabic, empty capsules, free durian extracts, non-encapsulated, and encapsulated extracts depending on the durian component source. **(A)** durian seed, **(B)** durian pulp, **(C)** durian peel.

Figure 3 shows the thermograms of the encapsulating agents, the free extracts, empty capsules, and the encapsulated extracts obtained from DSC. The polydextrose used in this study had an a_w_ of 0.2. Based on Figure 3, polydextrose exhibited a first endothermic peak at 67.77 °C, corresponding to the glass transition, reported with a_w_ values between 0.11 and 20 (56, 57). The second endothermic peak around 120–140 °C can be attributed to its melting point (58). Gum arabic exhibits a broader endothermic peak between 45 and 185 °C due to its glass transition temperature, reported to be around 56 to 150 °C depending on the purity of the gum arabic (59, 60).

The Dse, Dpu, and Dpe extracts in their free form showed a sharp endothermic peak at 110 °C, attributed to the melting of the crystalline structures of the extracted compounds. This effect is followed by a series of exothermic and endothermic peaks, culminating in a final exothermic peak at 170 °C, indicating the melting and degradation of the different compounds as temperature increases (61). The empty capsule (without durian extracts) curves showed melting behavior, with an onset temperature of 71.6 °C and a peak at 104.6 °C, but no sharp exothermic peak. For the capsules containing durian extracts, all Dse, Dpu, and Dpe curves exhibited onset and peak temperatures similar to those of the empty capsules. These findings indicate that encapsulation protects the compounds from thermal degradation in the temperature range used in this study. Quantitative comparison of DSC parameters reveals that encapsulation modified the thermal behavior of the extracts. The free Dse extract showed a sharp endothermic peak at 118.07 °C (integral: −457.94 mJ, normalized: −54.52 Jg^−1^), whereas the Dse capsules exhibited a broader endothermic event with peak at 115.85 °C (integral: −357.88 mJ, normalized: −43.62 Jg^−1^). The reduction in enthalpy (~20%) and broadening of the endothermic transition in the capsules indicate that the crystalline structure of the phenolic compounds was partially disrupted during encapsulation, resulting in a more amorphous state with improved thermal stability. Similarly, the Dpu free extract displayed an endothermic peak at 121.55 °C (integral: 13.37 mJ, normalized: 1.78 Jg^−1^), while Dpu capsules showed a shifted peak at 105.99 °C with substantially higher enthalpy (integral: −256.38 mJ, normalized: −36.63 Jg^−1^). The Dpe extract exhibited a peak at 135.81 °C (integral: −301.77 mJ, normalized: −49.47 Jg^−1^), and Dpe capsules showed a peak at 104.74 °C (integral: −189.92 mJ, normalized: −26.38 Jg^−1^) (Supplementary Tables S2–S4). The consistent shift in peak temperature and enthalpy across all encapsulated samples compared to their free extract counterparts confirms that the gum Arabic–polydextrose matrix creates molecular interactions that prevent the sharp crystalline melting observed in free extracts.

The absence of sharp exothermic peaks in the capsule thermograms, in contrast to the multiple exothermic events observed in free extracts above 170 °C, further supports the protective role of encapsulation against thermal degradation within the temperature range studied (−4 to 200 °C). These quantitative DSC findings provide robust evidence that the gum Arabic–polydextrose wall system effectively modifies the thermal behavior of durian bioactive extracts, enhancing their suitability for food processing applications involving heat treatment.

### 3.8 *In vitro* digestion of microcapsules

Table 4 shows the results for the TPC (mg GAE/200 mg) capsules before and after digestion through all three phases. The TPC value at the end of digestion is the phenolic content released from the microcapsule matrix and available for intestinal absorption, known as bioaccessibility (62). The bioaccessibility was calculated using the following formula:

$$\text{Bioaccessiblitiy}\;(\%)=(\begin{array}{l} \text{phenolic content released after the}\;\\ \text{gastrointestinal digestion}/\\ \text{phenolic content in the extract} \end{array})Χ100$$

**Table 4:** Total phenolic content (mg GAE eq./200 mg in Dse, Dpu, and Dpe) microcapsules before and after all phases of digestion (oral, gastric, intestinal).

	Before	After	Bioaccessibility (%)	P-value
Dse	8.3 ± 0.0*	8.1 ± 0.8	99.7	0.72
Dpu	1.4 ± 0.2	0.9 ± 0.1	69.8	0.04
Dpe	3.0 ± 0.1	2.5 ± 0.2	83.1	0.01

Dse = durian seed, Dpu = durian pulp, Dpe = durian peel. *Standard deviation from the mean.

It was observed that the phenolic content from Dse, Dpu, and Dpe capsules was readily available for intestinal absorption, as indicated by percent release ranging from 69.9 to 99.7% across the durian fruit components. The high accessibility of phenolic compounds after 120 min of digestion at the intestinal phase indicates that the polysaccharides and polydextrose can form protective complexes (63). Gum arabic, with its ramified structure and film-forming properties mentioned earlier, provides exceptional protection for the core material during digestion (64, 65). In addition, polydextrose, a complex polymer of glucose, is resistant to digestion; therefore, it serves as a prebiotic when fermented by the microbiota (66). Furthermore, polydextrose, as a wall material, can add benefits such as low a_w_ and moisture content and greater solubility (67). Therefore, the phenolic content from Dse, Dpu, and Dpe capsules will be accessible for intestinal absorption.

The markedly higher bioaccessibility of Dse capsules (99.7%) compared to Dpu (69.8%) and Dpe (83.1%) may be related to their distinct phenolic profiles. Dse extracts are rich in procyanidins (procyanidin B1: 1516.8 mg/L), catechin (593.7 mg/L), and epicatechin (527.5 mg/L), which are flavanol monomers and oligomers known for their relatively high stability under gastrointestinal pH conditions and susceptibility to enzymatic release from polysaccharide matrices. In contrast, Dpu extracts contain higher proportions of ferulic acid (720.8 mg/L), a hydroxycinnamic acid that can form ester bonds with cell wall polysaccharides, potentially reducing its release during *in vitro* digestion. The intermediate bioaccessibility of Dpe capsules (83.1%) is consistent with their mixed phenolic profile containing both highly bioaccessible flavanols and more resistant esterified phenolic acids.

Even under identical conditions of microencapsulation and in vitro assay, differences in % bioaccessibility of phenolic content after the intestinal phase may be due to variations in the intrinsic phenolic composition and concentration among durian components (Table 2). According to Quatrin et al., hydrolyzable tannins and flavonols from Jaboticaba peel powder had a greater bioaccessibility than anthocyanins (68). The amount of phenolic compounds during in vitro digestion can increase or decrease based on the digestive phase, as previously illustrated in various studies. For example, chlorogenic acid was found to be bioaccessible during the gastric phase due to absorption through the gastric mucosa. In contrast, quinic acid, being an esterified compound, was resistant to pH changes and to enzymes, making it difficult to be absorbed in the gastric and intestinal phases (69, 70). The bioaccessibility of flavonoids and anthocyanins from grapes was found to be increased during gastric digestion (71). A thorough study of the bioaccessibility of different classes of phenolic compounds in durian fruit components at each phase of digestion will shed further light on their implications as food additives.

## 4 Conclusion

Phenolic extracts from the seed, peel, and pulp of durian fruit represent a promising source of phenolic content and antioxidant activity. Notably, the total phenolic content, antioxidant activity, phenolic profile, and bioaccessibility of these extracts vary depending on the specific fruit component. The combination of gum arabic (53.1%) and polydextrose (46.9%) proved effective in preserving bioactive extracts and significantly enhancing their bioaccessibility following the intestinal phase of in vitro digestion. These microcapsules were thermostable over a wide temperature range (−4 to 200 °C), indicating their potential suitability as food additives for various food processing applications. These findings suggest that the optimal microencapsulation condition identified in this study is well-suited for durian bioactive extracts, offering the potential for improved delivery and utilization in functional food and nutraceutical applications. Moreover, the extraction of phenolic compounds and their microencapsulation will enhance the value of durian waste. In addition, durian waste can still be used to produce biochar or activated carbon after phenolic compounds are extracted. Future directions include assessing the bioavailability of phenolic compounds released from microcapsules, conducting sensory analysis, and conducting *in vivo* digestion tests with food products enriched with the encapsulated phenolic compounds.

## Data Availability

The raw data supporting the conclusions of this article will be made available by the authors, without undue reservation.
